# Set fire to the gall: Can the gall protect the galling weevil from fire?

**DOI:** 10.1002/ecy.70083

**Published:** 2025-05-07

**Authors:** Jean Carlos Santos, Henrique Venâncio, Guilherme Ramos Demetrio, Wanessa Rejane de Almeida, Walter Santos de Araújo, Pablo Cuevas‐Reyes

**Affiliations:** ^1^ Departamento de Ecologia Universidade Federal de Sergipe São Cristóvão Sergipe Brazil; ^2^ Programa de Pós‐Graduação em Entomologia, Faculdade de Filosofia, Ciências e Letras de Ribeirão Preto Universidade de São Paulo Ribeirão Preto São Paulo Brazil; ^3^ Programa de Pós‐Graduação em Ecologia & Conservação Universidade Federal de Sergipe São Cristóvão Sergipe Brazil; ^4^ Laboratory of Plant Ecology, U. E. Penedo, Campus Arapiraca Federal University of Alagoas Penedo Alagoas Brazil; ^5^ Departamento de Biologia Geral Universidade Estadual de Montes Claros Montes Claros Minas Gerais Brazil; ^6^ Laboratorio de Ecología de Interacciones Bióticas Universidad Michoacana de San Nicolás de Hidalgo, Ciudad Universitaria Morelia Michoacán México

**Keywords:** Brazilian savanna, Cerrado, epidermis, gall‐inducing insect, insect gall, plant–insect interactions, protection

Fire is one of the main causes of habitat disturbance that negatively affects biodiversity, causing changes in vegetation structure and plant biomass as well as disruptions in plant–animal interactions in many terrestrial ecosystems (Grau‐Andrés et al., 2024; Kelly et al., 2020). The occurrence of fire has deep consequences for the ecology and evolution of insect herbivores (Koltz et al., 2018), and it also has direct consequences on these insect assemblies because of the loss of food resources and shelter (Knight & Holt, 2005; New, 2014). The direct and immediate effects of fire on individual insects and their populations, including cremation and lethal heat exposure, can be severe and lead to large‐scale mortality (New, 2014). Particularly in less mobile insect species and ontogenetic stages such as eggs, larvae, and pupae, it is possible to expect that the effects of fire will be more severe (Koltz et al., 2018). However, the effects of fire on insects are poorly understood (Arruda et al., 2018), and, according to New (2014), few terrestrial or aquatic insects are completely immune to the effects of fire (see Bieber et al., 2023).

Many insects induce galls, tumors with atypical plant tissue growth, on their host plants. These changes in plant tissues are attributed to hyperplasia and/or hypertrophy of plant cells induced by specific stimuli from a female insect while laying eggs and/or by offspring while feeding on plant tissues (Giron et al., 2016). The resulting galls provide shelter, food, and protection from natural enemies during larval development (Giron et al., 2016). For example, thick gall wall epidermis can provide a rigid barrier that confers protection to the gall‐inducing insect larvae against natural enemies and abiotic stressors such as high temperatures and low water availability (Stone & Schönrogge, 2003). Gall‐inducing insects are highly diverse in the Neotropics, especially in the Cerrado (Brazilian Savannah) (Fernandes & Santos, 2014), which is a fire‐adapted ecosystem (Durigan, 2020) with a high incidence of anthropogenic fires (Pivello, 2011).

Here, we report the first case of galls conferring protection against fire to the gall‐inducing Boheman weevil *Collabismus clitellae* Boheman (Coleoptera: Curculionidae). The Boheman weevil induces woody galls on *Solanum lycocarpum* St. Hil. (Solanaceae). *S. lycocarpum*, locally known as “*lobeira*” (“wolf's fruit”), is a shrub that can reach up to 3 m in height and is abundant throughout the Cerrado. *C. clitellae* females oviposit on new shoots, resulting in the induction of multi‐chambered galls that harbor high densities of larvae (1–70 chambers per gall, but with only one individual in each chamber) (Souza et al., 1998). The galls of *C. clitellae* vary in size, from 1.3 to 16.9 cm in length and from 0.6 to 4.7 cm in width, and volume, from 0.3 to 140 cm^3^ depending on the number of eggs deposited (Souza et al., 1998).

The study was conducted at Nova Monte Carmelo Farm (52,000 ha, Minas Gerais, Brazil; 18°57′ S, 47°43' W), where an extensive fire occurred in August 2012, affecting both the *Eucalyptus* forests and the natural areas of the Cerrado (Figure 1; Appendix S1: Figure S1). The fire lasted 24 h and reached at least 30% of the protected area. One day after the fire (August 16, 2012), we randomly sampled 83 galls from 40 *S. lycocarpum* plants, 52 of which were from 28 plants in burnt areas and 31 from 12 plants in unburnt (control) areas (Appendix S1: Figure S2). All galls were dissected in the laboratory using pruning shears to determine the number of weevils and the survival of *C. clitellae* larvae and pupae by looking for activity. The percentage of surviving weevils was calculated as the ratio of the number of survivors to the total number of weevils in each gall.

**FIGURE 1:**
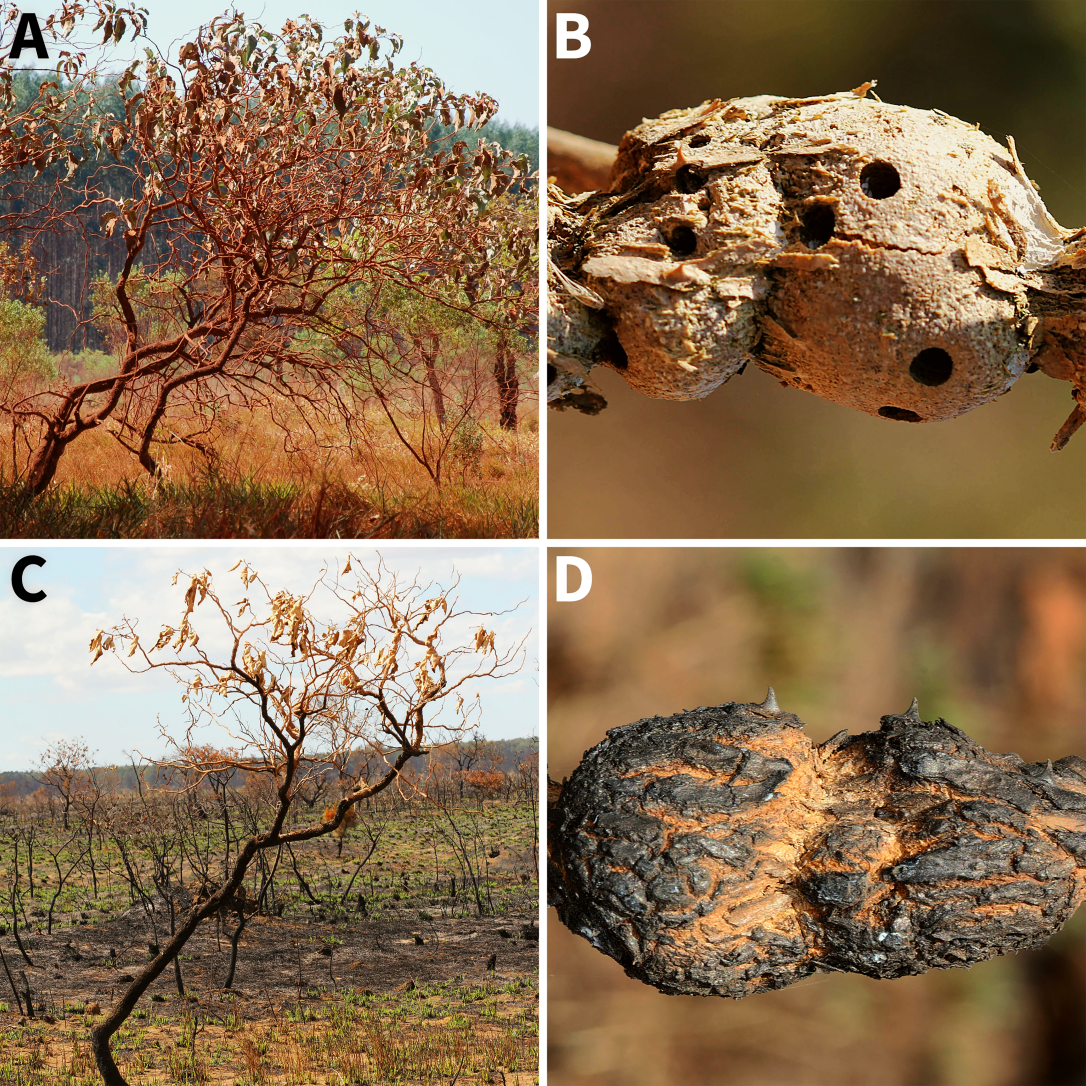
Host plant *Solanum lycocarpum* St. Hil. (Solanaceae) and the gall of the weevil *Collabismus clitellae* Boheman (Coleoptera: Curculionidae), unburnt (A, B) and burnt (C, D). Photo credits: J.C. Santos.

To determine the traits of burnt plants and galls that affected the survival of galling weevils, we measured the following: (1) plant height and (2) gall height above the ground as a measure of fire proximity; (3) length, width, and volume of galls; (4) gall shape index, calculated using the formula “gall length/gall width”; (5) weevil density, estimated as the number of larvae and pupae per gall volume; and (6) gall epidermis thickness. Gall volume (in cubic millimeters) was calculated using cylinder volume formula: $\text{Volume}=\pi hr^{2}$, where *h* is gall length (in millimeters) and *r* is half of gall width. We also investigated the effects of these variables on *C. clitellae* survival following a fire event. To do so, we divided the burnt galls into two groups: “Full Survival” (FS = burnt galls with all weevils surviving) (*N* = 20 galls) and “Galls with Mortality” (GM = burnt galls in which at least one weevil did not survive) (*N* = 32 galls) (see Santos et al., 2025). We used generalized linear mixed models (GLMM) to test the protective effect of galls on the survival of *C. clitellae*. These models used gall traits along with their interaction with the fire condition (burnt or unburnt) as predictors of the percentage of weevil survival, which was used as a response variable. For all the models, we used the gall nested in the plant as a random factor. These models were built with the function glmmTMB of the glmmTMB package (Brooks et al., 2017) using the beta distribution family. We also used the function rsquared of the piecewiseSEM package to calculate the conditional *R* ^2^, which corresponds to the total variance explained by the fixed factors (Lefcheck, 2016). We also built GLMMs to test for differences in plant and gall traits between burnt gall groups (FS and GM). In each model, the fire condition (burnt or unburnt) was considered as a fixed variable and the gall nested in the plant as a random factor. The Gaussian family was used in all models, except for insect gall density, where the Poisson family was applied.

We observed that unburnt galls were parasitized by a natural enemy, an unidentified parasitoid species (Hymenoptera: Braconidae), which caused a mortality rate of ~42% of weevils in unburnt plants. We then compared the average weevil survival rates of these unburnt galls with burnt galls, where the weevils were killed only by fire, with none of them parasitized (Figure 2). The observed disparities in parasitoidism rates between burnt and unburnt areas suggest that fire may reduce the trophic complexity associated with galls, potentially eliminating higher and more specialized trophic levels (Batista et al., 2023). An additional hypothesis we proposed is that parasitoids may be selecting areas with no occurrence of fire and/or less frequent fire events to parasitize insect galls, potentially conferring an adaptive advantage. However, both hypotheses require further investigation and testing.

**FIGURE 2:**
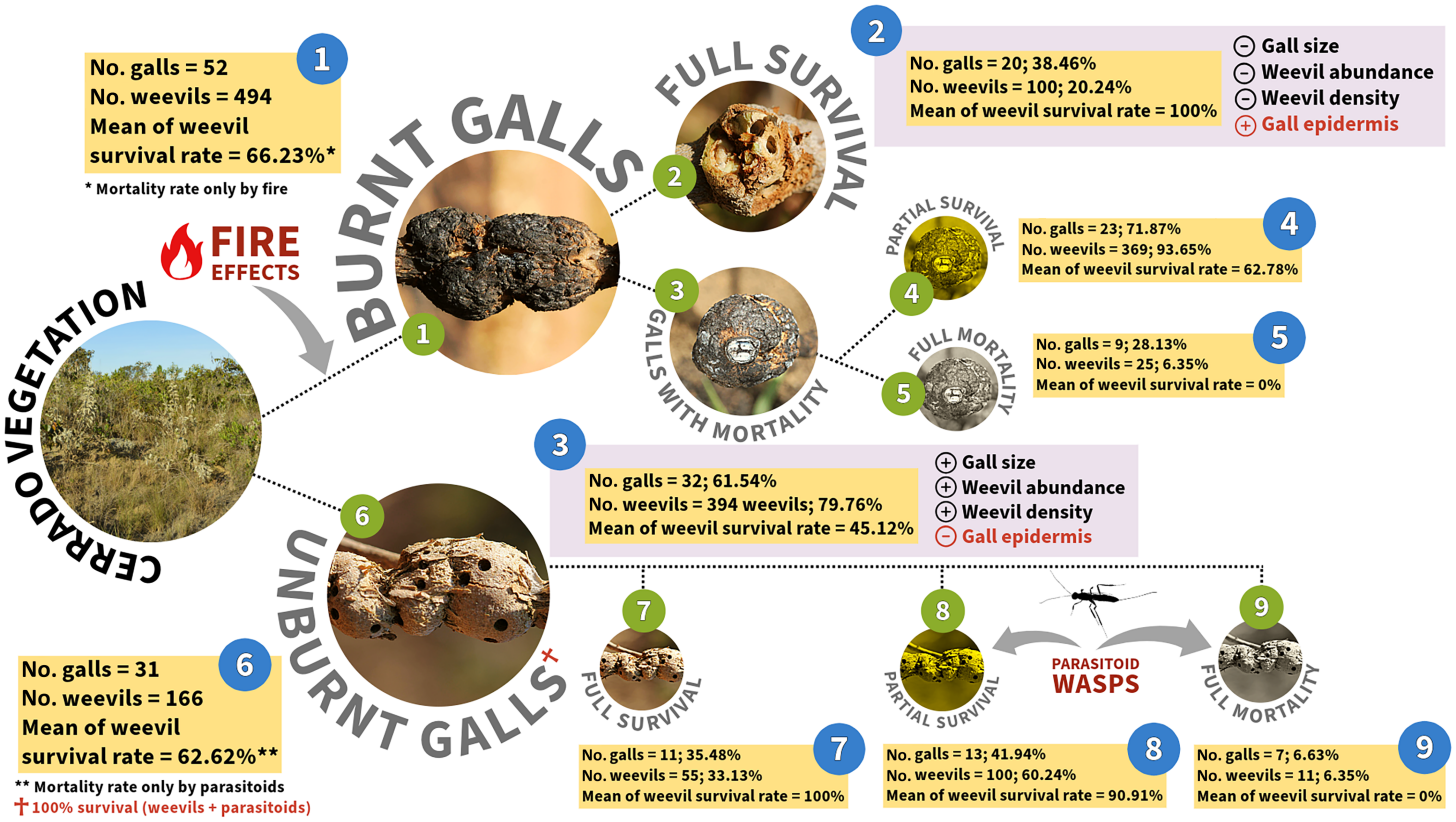
Organizational chart comparing specific variables of *Collabismus clitellae* (Coleoptera: Curculionidae) galls in *Solanum lycocarpum* (Solanaceae) that survived and perished owing to fire effects in the Brazilian Cerrado. Comparisons: 1 versus 6 (unburnt vs. burnt galls), 2 versus 3 (galls with full survival vs. galls with mortality), and 4 versus 5 (galls with mortality/partial survival versus galls with mortality/full mortality). Photo credits: J.C. Santos.

Our model used a beta distribution in which the response variable was modeled on a transformed scale using a logit link function. Therefore, the estimated coefficient of 3.296 for unburnt areas (Appendix S1: Table S2) represents the effect of the absence of fire on weevil survival rate on a logit scale. To interpret this effect in terms of weevil survival rate, we exponentiated the coefficient, obtaining an odds ratio (OR) of approximately 27. In this sense, weevils from unburnt areas had a survival rate 27‐fold higher than that of those from burnt areas (Appendix S1: Table S2). This finding suggests that fire is a significant cause of mortality in burnt galls.

We disaggregated the results to better visualize the effects of fire on the galls (Figure 2). Overall, the mean weevil survival rate for burnt galls was 66%, suggesting that the galling weevils tolerated the fire (Figure 2, box 1). Thirty‐eight percent of the burnt galls contained all weevils alive and remained largely intact postfire, despite scorching of the epidermis of the host plant and galls (Figure 2, box 2). The remaining 62% of burnt galls were categorized as “galls with mortality” (Figure 2, box 3), resulting in two groups: (1) a group in which galls exhibited partial weevil survival, with survival rates ranging from 28.6% to 88.24% (Figure 2, box 4), and (2) a group with zero weevil survival (Figure 2, box 5). The control population of galls unexposed to fire demonstrated mortality caused solely by natural enemies. Specifically, parasitoids were responsible for the mortality of approximately 37% of the gall inducers, with 62.62% of the inducers surviving (Figure 2, box 6). Thirty‐five percent of the galls in this area were unaffected by parasitoids (Figure 2, box 7). The highest survival rate (90.91%) of weevils occurred in approximately 42% of galls (Figure 2, box 8), and total mortality occurred in approximately 7% (Figure 2, box 8).

When comparing burnt and unburnt areas, we found that plant height was not a good predictor of weevil survival (χ^2^ = 1.86, df = 3, *p =* 0.6). Similarly, gall height (χ^2^ = 5.23, df = 3, *p =* 0.15), length (χ^2^ = 2.66, df = 3, *p =* 0.44), width (χ^2^ = 4.91, df = 3, *p =* 0.17), and volume (χ^2^ = 3.05, *p* = 0.081) were not good predictors of the survival of weevils, independent of the fire condition (Appendix S1: Table S1). However, gall epidermis thickness presented an interesting relationship with beetle survival. In the burnt area, thicker gall epidermis was related to higher survival rates, whereas in the unburnt area, thicker gall epidermis was related to lower survival rates (Appendix S1: Figure S3). In this model, gall epidermis thickness and the situation of the area showed a conditional *R* ^2^ = 0.35, indicating a moderately strong relationship with survival rates. This suggests that gall epidermis thickness can act as protective factors against fires in immature weevils.

However, contrary to our expectations, burnt galls without mortality were smaller (51.80 ± 3.95 mm) and had fewer individuals per gall (5.00 ± 1.17 mm) than burnt galls with weevil mortality (size: 70.36 ± 5.68 mm, χ^2^ = 5.82, *p* = 0.016 and density: 12.31 ± 2.27, χ^2^ = 9.19, *p* = 0.002; Figure 2, boxes 2 and 3). The epidermis layer of burnt gall with alive weevils was larger compared with burnt galls with weevil mortality, indicating the protective role of gall tissues against the fire effects (FS = 2.13 ± 0.19 mm and GM = 1.60 ± 0.14 mm; χ^2^ = −5.53; *p* = 0.019) (Figure 3; Appendix S1: Table S2). Finally, the shape gall index did not differ significantly between groups (Appendix S1).

**FIGURE 3:**
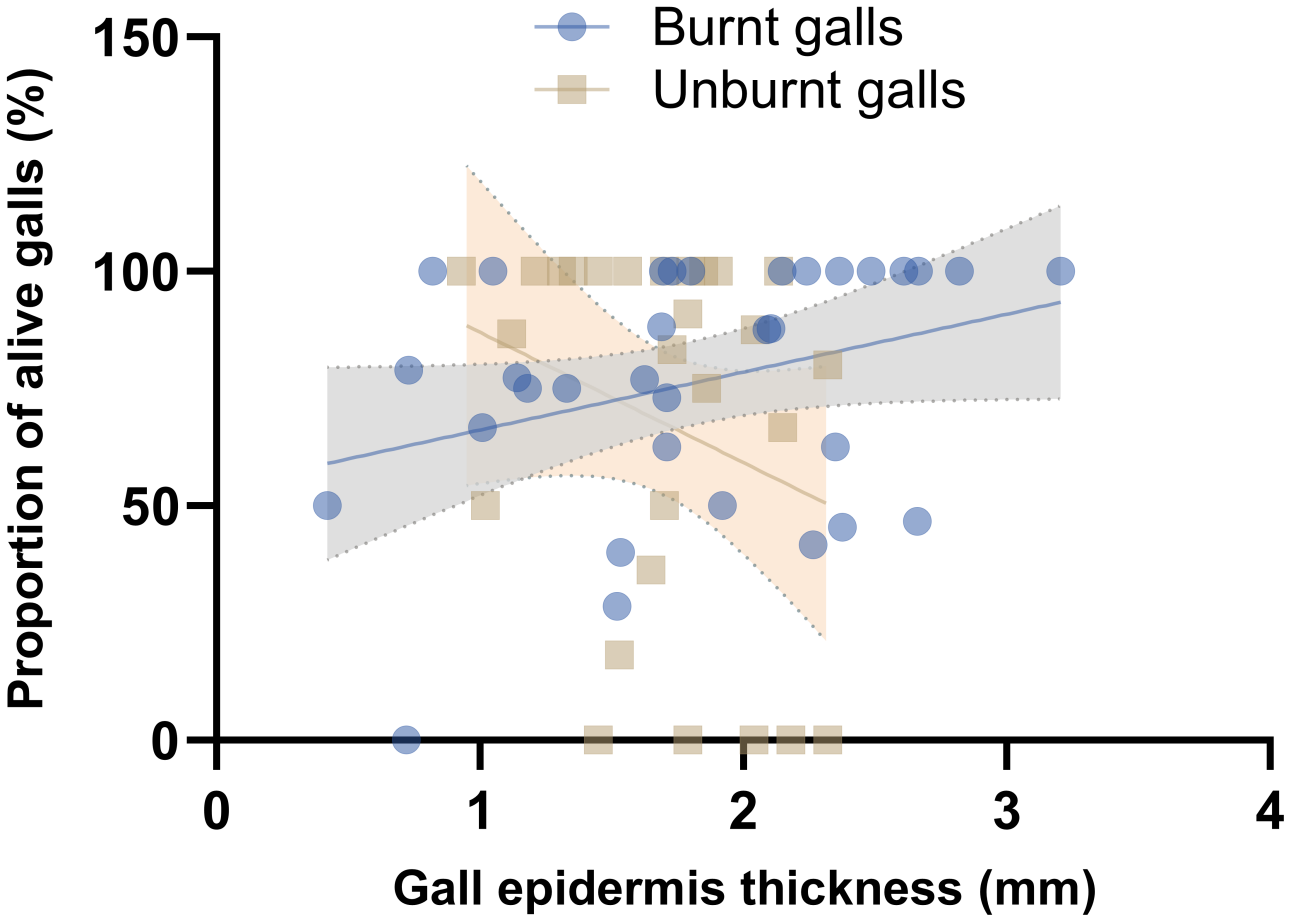
Relationship between the proportion of *Collabismus clitellae* Boheman (Coleoptera: Curculionidae) galls alive and gall epidermis thickness (in millimeters) of *Solanum lycocarpum* St. Hil. (Solanaceae) in the burnt and unburnt areas. The results showed a significant interaction between the epidermis layer and fire condition, indicating a positive effect of gall survivorship and gall epidermis thickness affected by fire, and decreased gall survivorship with unburnt epidermis increment.

Previous studies on galls under fire disturbance have addressed the effects of changes in the resource availability of host plants on the postfire gall community structure (Andrade et al., 2019; Cronin et al., 2020). To the best of our knowledge, few studies have focused on the direct effects of fire on galls and their occupants. For instance, Fay and Samenus (1993) demonstrated that fire caused insect mortality in 100% of gall wasps (Hymenoptera: Cynipidae), incinerating shoot galls and their inhabitants. Our study provides new insights by demonstrating that the gall‐inducing weevil *C. clitellae* can be tolerant of fire, suggesting that gall traits such as gall epidermis thickness play an important role in protecting gall‐inducing weevils against fire in the Cerrado ecosystem.

In addition, weevil survival is associated with life‐history traits such as gall size and the number of weevils per gall. Our results showed that FS were smaller and had lower densities of weevils than GM. Souza et al. (2001) showed that the number of chambers within the galls induced by *C. clitellae* increased with gall size. Moreover, increasing gall size also increases weevil density and reduces adult size and developmental rates, probably because of resource limitations. Therefore, considering that each gall is inhabited by several weevils, when there is a strong ecological disturbance (e.g., fire), galls with fewer weevils suffer less damage from fire because of their lower density. However, despite our limited data, more information on gall traits and fire is required to provide a mechanistic understanding of gall survival under fire stress.

Gall‐inducing insects are sophisticated endophytic herbivores that are capable of manipulating and reorganizing host plant tissues to create a physical structure, the gall, within which larvae feed and grow (Giron et al., 2016). This gall‐inducing habit can provide a microclimate and protection for the larva from natural enemies and abiotic factors (e.g., Giron et al., 2016; Price et al., 1987; Stone & Schönrogge, 2003). A possible adaptive explanation is that the epidermal tissue may have a fundamental role in gall protection (attenuating or preventing fire effects), probably by reducing direct exposure to the flames. Indeed, we showed that the gall epidermis was thicker in FS than in GM, indicating that an increase in the thickness of the epidermis increases the survival of weevils. In addition, we observed that the inner tissues of the galls of *C. clitellae* were lignified, which could provide more protection from fire to weevils than non‐lignified tissues (e.g., leaves, which are habitats for most gall‐inducing insects).

An additional finding regarding the gall shape index further strengthens the role of epidermis thickness in fire resistance. Our results demonstrate that variations in gall morphology, whether spherical or elongated, did not significantly influence weevil survival. We hypothesized that spherical galls would exhibit greater fire resistance than elongated galls; however, this hypothesis is not supported by our findings. As mentioned above, the epidermis appears to function as a protective barrier against fire damage, acting as a thermal buffer mediated by non‐nutritive tissues of galls (the microenvironment hypothesis; see Giron et al., 2016; Price et al., 1987; Stone & Schönrogge, 2003) irrespective of the gall shape. While morphological interspecific diversity in galls has been linked to defense mechanisms against abiotic stress and/or natural enemies (Giron et al., 2016; Price et al., 1987; Stone & Schönrogge, 2003), the role of intraspecific variations in gall shape in mitigating abiotic disturbances, such as fire, remains poorly understood. This highlights the need for further investigation of how gall traits contribute to resilience under varying environmental pressures.

In neotropical savanna woody species, thick bark is a successful fire protection trait in ecosystems where fires are intense (Dantas & Pausas, 2013). In these ecosystems, fire regimes select woody species with thicker bark because of their heat insulation properties, which protect plant tissues (e.g., meristematic tissues, phloem, and xylem) from flames and high temperatures (Pausas, 2015). Analogically, we observed a similarity between the thick bark and gall epidermis thickness of *S. lycocarpum* induced by *C. clitellae*, and we demonstrated that thicker gall walls provide effective protection for *C. clitellae* against fire damage, as analogous to thick bark providing protection for plants. Despite our limited data, we suggest further investigations involving histological studies to determine the effects of fire on stem galls and host plant tissues. We also propose that future studies should assess this scenario using an evolutionary ecological approach, given that insect herbivores co‐evolve with their host plants (Leimu et al., 2012; Maron et al., 2019), and that natural selection may exert a significant influence by selecting induction of gall thickness and size as a function of natural enemies, such as predators and/or parasitoids (see Abrahamson et al., 1989; Weis & Abrahamson, 1985). The results of our investigation indicate selective pressure from fire, favoring smaller and thicker gall phenotypes (Laine & Tylianakis, 2024). Finally, our findings prompted intriguing questions: Are weevils in stem galls fire tolerant the same way as the host plant, with bark or gall epidermis thickness a mechanism to deal with this natural, predictable, and recurrent disturbance in the Cerrado? If so, does the protection depend on the host plant? Alternatively, weevils in galls may have evolved fire tolerance independently of the host plant. Furthermore, there are inquiries of ecological and evolutionary nature, such as whether inhabiting fire‐stressed environments like the Cerrado influences gall size and whether natural selection exerts pressure on this trait. These unresolved questions present opportunities for future research to explore new avenues for investigation.

## CONFLICT OF INTEREST STATEMENT

The authors declare no conflicts of interest.

## Supporting information


Appendix S1:

## Data Availability

Data (Santos et al., 2025) are available in Zenodo at https://doi.org/10.5281/zenodo.14934224.
