# Supplementary material for: Set fire to the gall: Can the gall protect the galling weevil from fire?
Source: Ecology. 2025 May 7;106(5):e70083. doi: 10.1002/ecy.70083 (PMC12056535; doi:10.1002/ecy.70083)
Supplement: Supplementary file 1 — Appendix S1: [file ECY-106-e70083-s001.pdf]

APPENDIX S1

Set fire to the gall: Can the gall protect the galling weevil from fire?

Jean Carlos Santos, Henrique Venâncio, Guilherme Ramos Demetrio, Wanessa Rejane de Almeida, Walter Santos de Araújo, and Pablo Cuevas-Reyes

Journal: Ecology

Figure S1. Characterization of the study area: (A) unburnt and (B) burnt areas of the Cerrado. (C) Grass, (D) vegetative parts, and (E) trunks affected by fire. (F) Hemipteran charred by fire. Photo credits: J.C. Santos.

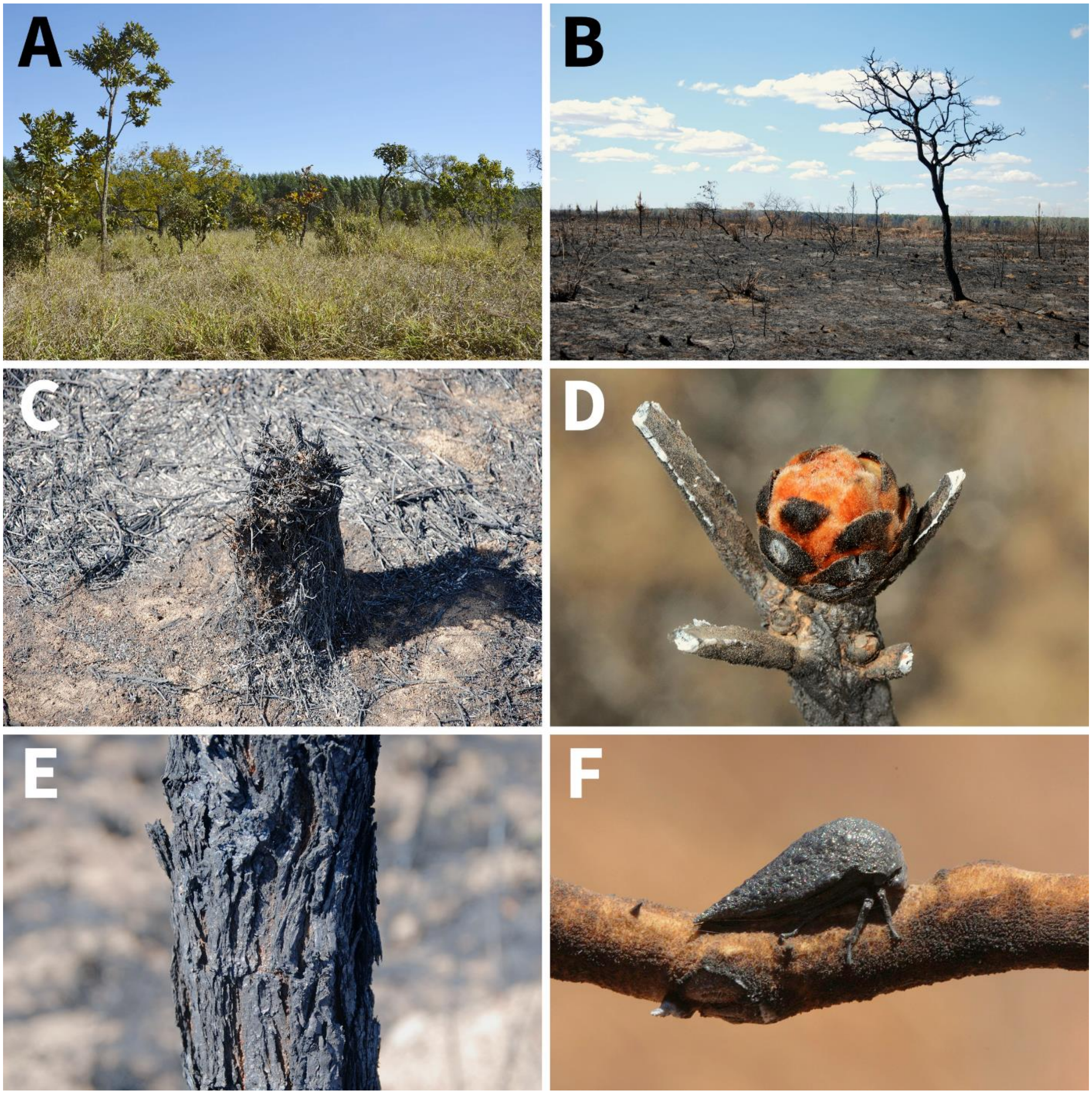

Figure S2. Examples of burnt galls induced by the weevil *Collabismus clitellae* Boheman (Coleoptera: Curculionidae). Photo credits: J.C. Santos.

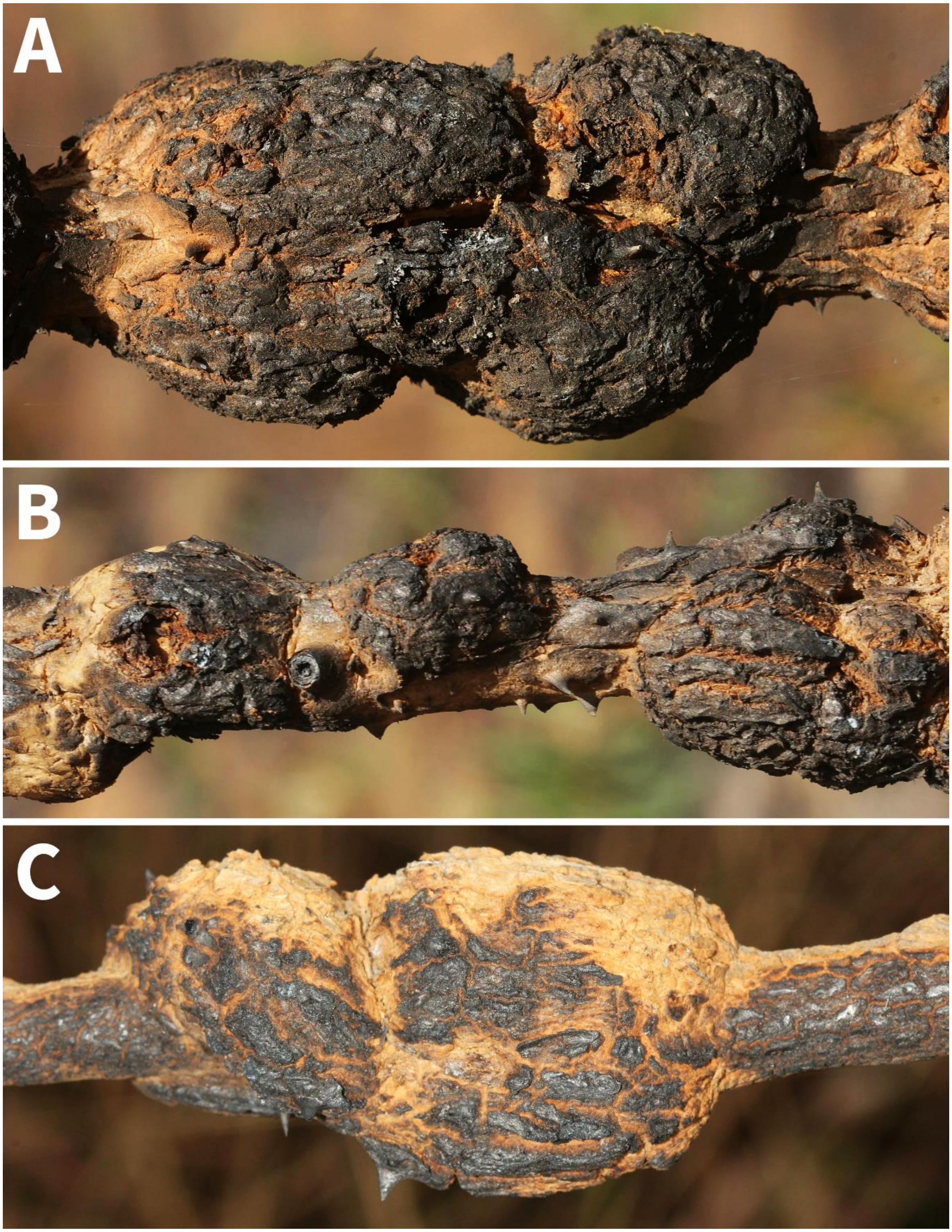

Figure S3. A cut in a gall exposed the outer and inner tissues and larval chamber of the galling weevil, *Collabismus clitellae* (Coleoptera: Curculionidae). (B) A cut into a gall containing gall tissues and a weevil in the larval chamber. Photo credits: J.C. Santos.

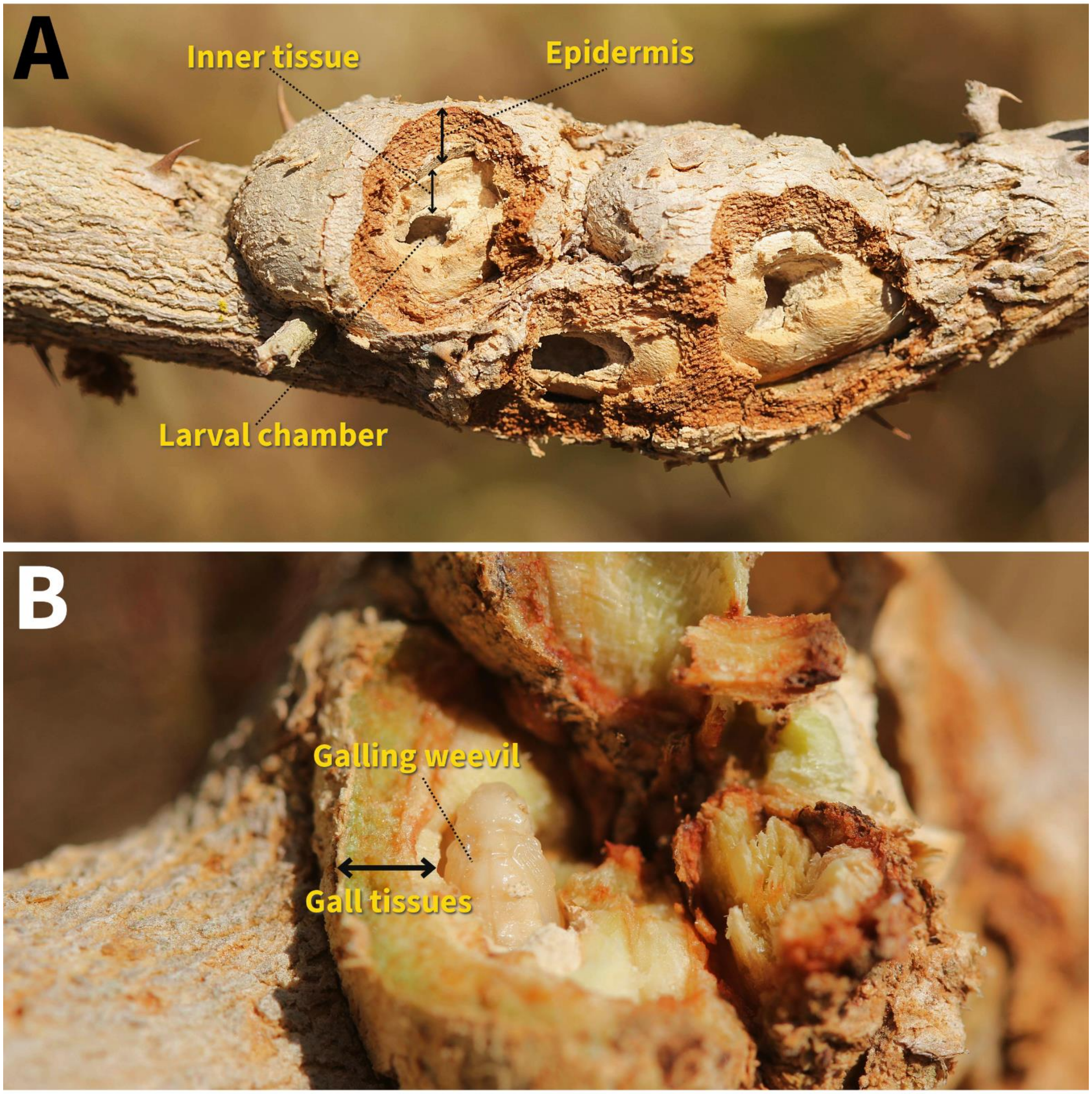

Table S1. Comparison (mean ± SE) of some variables between unburnt and burnt galls of *Collabismus clitellae* (Coleoptera: Curculionidae) in *Solanum lycocarpum* (Solanaceae). In addition, we statistically compared galls that survived and those that died in the burnt gall group. The "Full Survival" (FS) represents a subset of burnt galls where all weevils survived and were not affected by the fire, while the "Galls with Mortality" (GM) represents a subset of burnt galls where at least one weevil did not survive owing to fire effects. Statistically significant values are in bold.

**Variables**	**Unburnt Galls**	**Burnt Galls**		**χ^2; *p*-value; distribution type**
		**Full Survival**	**Galls with Mortality**	
Plant height (m)	3.24 ± 1.93 (N=31)	1.21 ± 0.13 (N=20)	1.33 ± 0.12 (N=32)	χ^2 = 0.78; *p* = 0.489 (n.s.); Gaussian
Gall height above ground (m)	0.653 ± 0.096 (N=31)	0.781 ± 0.067 (N=20)	0.892 ± 0.083 (N=32)	χ^2 = 1.18; *p* = 0.277 (n.s.); Gaussian
Gall length (mm)	58.02 ± 5.33 (N=31)	51.80 ± 3.95 (N=20)	70.36 ± 5.68 (N=32)	**χ^2 = 5.82; *p* = 0.016; Gaussian**
Gall width (mm)	30.97 ± 1.65 (N=31)	27.58 ± 1.85 (N=20)	30.72 ± 1.36 (N=32)	χ^2 = 2.04; *p* = 0.153 (n.s.); Gaussian
Gall volume (mm^3)	3,084 ± 378 (N=31)	3,552 ± 390 (N=20)	2,383 ± 279 (N=32)	χ^2 = 3.05; *p* = 0.081 (n.s.); Gaussian
Weevil abundance (nº of larvae/pupae per gall)	5.36 ± 0.86 (N=31)	5.00 ± 1.17 (N=20)	12.31 ± 2.27 (N=32)	**χ^2 = 9.19; *p* = 0.002; Gaussian**
Weevil density (nº of larvae/pupae per mm^3)	$17.83^{-4} \pm 1.63^{-4}$ (N=31)	$18.85^{-4} \pm 3.14^{-4}$ (N=20)	$31.63^{-4} \pm 3.87^{-4}$ (N=32)	**χ^2 = 6.11; *p* = 0.013; Beta**
Gall epidermis thickness (mm)	1.70 ± 0.07 (N=24)	2.13 ± 0.19 (N=12)	1.60 ± 0.14 (N=20)	**χ^2 = -5.53; *p* = 0.019; Gaussian**
Shape gall index	1.86 ± 0.13 (N=31)	1.92 ± 0.14 (N=20)	2.34 ± 0.17 (N=32)	χ^2 = 1.58; *p* = 0.208 (n.s.); Gaussian

n.s. = no statistical difference

Table S2. Results of GLMMs evaluating the influence of fire conditions (burnt and unburnt), plants, and gall traits on the survival of *Collabismus clitellae* (Coleoptera: Curculionidae). We considered survival rate as the number of live *C. clitellae* per gall in relation to the total number of chambers as weevil survival. In each model, we included the weevil survival rate as the response variable and the fire condition of the area (burnt or not burnt) and its interaction with each plant (plant height) or gall trait (gall height, gall width, gall length, and gall epidermis thickness) as fixed variables. The gall ID nested in the plant ID was used as a random factor. We adopted a level of significance of 95% ($\alpha = 0.05$). Pr(>|z|) represents the probability (Pr) of obtaining a value as extreme as (or more extreme than) the observed z-value under the null hypothesis that the predictor variable estimate is zero. Statistically significant p values are shown in bold.

**Model**	**Source of variation**	**Estimate**	**Std. Error**	**z-value**	**Pr(>|z|)**
Survival rate ~ Gall epidermis thickness * Fire condition	Intercept	- 0.357	0.660	-0.541	0.588
	Gall epidermis thickness	0.585	0.338	1.732	0.083
	Fire Condition (unburnt area)	3.296	1.371	2.404	**0.0162**
	Gall epidermis thickness * Fire Condition (unburnt area)	-2.133	0.771	-2.764	**0.005**
